# Trigonelline hydrochloride attenuates silica-induced pulmonary fibrosis by orchestrating fibroblast to myofibroblast differentiation

**DOI:** 10.1186/s12931-024-02876-1

**Published:** 2024-06-15

**Authors:** Fengqin Zhang, Huihui Yue, Ruihan Dong, Jianhan He, Ling Zhou, Xinran Dou, lingling Wang, Pengdou Zheng, Zhenyu Mao, Xiaoyan Zhu, Yi Wang, Huiguo Liu, Huilan Zhang

**Affiliations:** Department of Respiratory and Critical Care Medicine, National Health Commission Key Laboratory of Respiratory Diseases, Tongji Hospital, Tongji Medical College, Huazhong University of Science and Technology, 1095 Jiefang Ave, Wuhan, 430030 China

**Keywords:** Trigonelline, Silicosis, Fibroblast, Pulmonary fibrosis

## Abstract

**Background:**

Silicosis represents a paramount occupational health hazard globally, with its incidence, morbidity, and mortality on an upward trajectory, posing substantial clinical dilemmas due to limited effective treatment options available. Trigonelline (Trig), a plant alkaloid extracted mainly from coffee and fenugreek, have diverse biological properties such as protecting dermal fibroblasts against ultraviolet radiation and has the potential to inhibit collagen synthesis. However, it’s unclear whether Trig inhibits fibroblast activation to attenuate silicosis-induced pulmonary fibrosis is unclear.

**Methods:**

To evaluate the therapeutic efficacy of Trig in the context of silicosis-related pulmonary fibrosis, a mouse model of silicosis was utilized. The investigation seeks to elucidated Trig's impact on the progression of silica-induced pulmonary fibrosis by evaluating protein expression, mRNA levels and employing Hematoxylin and Eosin (H&E), Masson's trichrome, and Sirius Red staining. Subsequently, we explored the mechanism underlying of its functions.

**Results:**

In vivo experiment, Trig has been demonstrated the significant efficacy in mitigating SiO_2_-induced silicosis and BLM-induced pulmonary fibrosis, as evidenced by improved histochemical staining and reduced fibrotic marker expressions. Additionally, we showed that the differentiation of fibroblast to myofibroblast was imped in Trig + SiO_2_ group. In terms of mechanism, we obtained in vitro evidence that Trig inhibited fibroblast-to-myofibroblast differentiation by repressing TGF-β/Smad signaling according to the in vitro evidence. Notably, our finding indicated that Trig seemed to be safe in mice and fibroblasts.

**Conclusion:**

In summary, Trig attenuated the severity of silicosis-related pulmonary fibrosis by alleviating the differentiation of myofibroblasts, indicating the development of novel therapeutic approaches for silicosis fibrosis.

**Supplementary Information:**

The online version contains supplementary material available at 10.1186/s12931-024-02876-1.

## Introduction

Silicosis is an occupational disease caused by long-term inhalation of crystalline silica, which is irreversible and ultimately fatal, characterized by pulmonary dysfunction, persistent lung inflammation and irreversible pulmonary fibrosis leading to respiratory failure and death [1, 2]. The Global Burden of Disease Study 2019 reports an increase in the number of new cases of silicosis from 84,400 in 1990 to 139,000 in 2019 across 204 countries and territories. During the same period, there were 15,100 to 12,900 deaths. The prevalence and incidence of silicosis have been rising, especially in developing countries, although it’s also a high-profile occupational health problem in developed countries [3–5]. Currently, only nintedanib and pirfenidone have been approved by the US Food and Drug Administration (FDA) to treat pulmonary fibrosis. However, there is still no standard treatment option for silicosis [6, 7]. The limited supply of donor lungs and the high expense of the procedure prevent lung transplantation to being widely utilized, despite the possibility of increasing survival. Therefore, it is imperative to find more effective drugs or components for treating silicosis.

Although the pathophysiology of silicosis is still not fully understood, research has indicated that a number of cells, including fibroblasts, epithelial cells, and macrophages, are implicated in the formation of silicosis fibrosis [8–11]. Upon inhalation of silica, alveolar macrophages continue the process of phagocytosis, exacerbating inflammation and releasing cytokines such as transforming growth factor-β1 (TGF-β1), which is essential for the activation and differentiation of fibroblasts into myofibroblasts. The process of differentiation, a defining characteristic of fibrotic diseases, results in the overproduction and accumulation of extracellular matrix (ECM) proteins like collagen, fibronectin, and elastin, ultimately leading to the development of pulmonary fibrosis [12]. Infiltrating monocytes may exert protective effects in silicosis by preventing fibroblast activation [13]. Furthermore, the activation and transdifferentiation of fibroblasts are facilitated by p53/PUMA, IRF4, miR-125a-5p, and ALKBH5, which exacerbates the development of silicosis fibrosis [14–17]. Conversely, miR-503, metformin, Apelin, and the CD44-RhoA-YAP pathway ameliorate silica-induced pulmonary fibrosis by inhibiting fibroblast activation and transdifferentiation [18–21]. In conclusion, the activation and differentiation of fibroblasts have been identified as the primary mechanism underlying the formation of silicosis fibers [22–24].Therefore, inhibition of fibroblast activation and differentiation is a viable strategy for the development of novel antifibrotic drugs for silicosis.

Trigonelline (Trig), a plant-derived alkaloid predominantly extracted from coffee and fenugreek, performed a multitude of biological activities according to extant research, such as antioxidant, anti-inflammatory, and anticancer. Additionally, Trig provides protection to dermal fibroblasts against UV-induced damage, by suppressing collagen synthesis, thereby alleviating renal fibrosis [25–31]. However, it’s unclear whether Trig inhibits fibroblast activation to attenuate silicosis-induced pulmonary fibrosis.

In this study, we explored the anti-fibrotic potential of Trig on silicosis in vivo and in vitro, along with its underlying mechanisms. We indicated that Trig may be potentially exert therapeutic benefits against silicosis-related fibrosis by inhibiting the TGF-β/Smad signaling pathway.

## Materials and methods

### Experimental materials

**Fig. 1:**
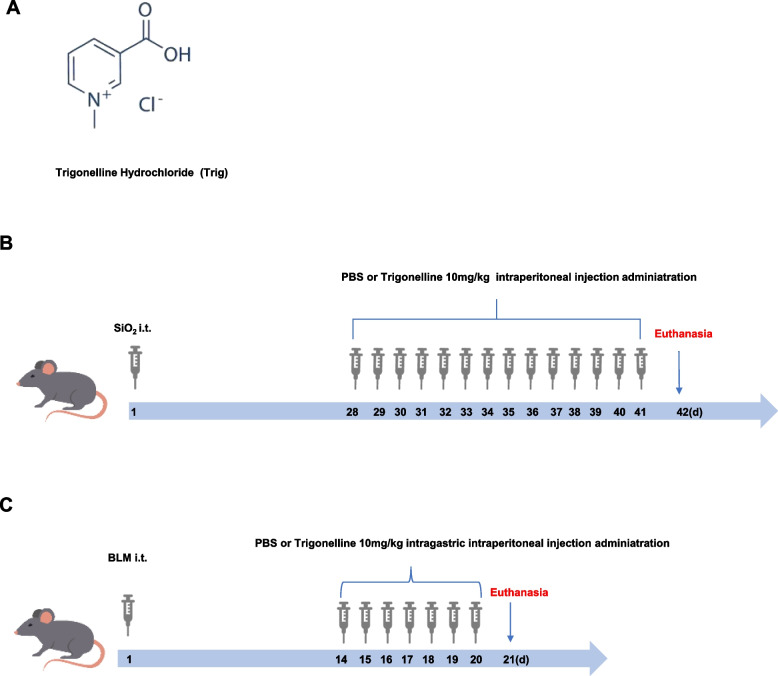
Silicon dioxide and BLM-induced mouse model administration times. **A** Chemical structure of trigonelline hydrochloride; (**B**) silica-induced pulmonary fibrosis model and administration time; (**C**) BLM-induced mouse pulmonary fibrosis model and administration time

High-sugar DMEM medium and fetal bovine serum were purchased from Gibco, USA; Trypsin was purchased from Wuhan Kerui Co., Ltd; Trizol was purchased from Takara, Japan; Reverse transcription and real-time fluorescence quantitative PCR kit was purchased from vazyme, Nanjing, China; RIPA lysis solution was purchased from Beyotime, Shanghai, China; PVDF membrane was purchased from Millipore, USA ECL luminescent solution was purchased from Google Bio; ThermoFisher incubator was purchased from ThermoFisher, USA; Biomicroscope was purchased from Olympus, Japan; Real-time fluorescence PCR instrument was purchased from Bio-Rad, USA; BLM was purchased from Pfizer; Recombinant human TGF-β1 was purchased from PeproTech, USA. Trigonelline Hydrochloride was purchased from Selleck, Shanghai, China (Fig. 1A).

### Experimental mice

The male, and of wild-type C57BL/6 background 8–10 weeks old, (Beijing Viton Lihua Laboratory Animal Technology Co.) were selected in this study. The animal breeding and experiments were conducted in the Specific Pathogen-Free Laboratory Animal Center in the Research Building of Tongji Hospital, Huazhong University of Science and Technology. The animal experiments were conducted in accordance with the principles of the National Institutes of Health and were approved by the Animal Research Committee of Tongji Hospital, Huazhong University of Science and Technology (TJH-202312005).

### Animal model

The SiO_2_-induced fibrosis model involved randomly dividing mice into four groups: (1) PBS + PBS group, (2) PBS + Trig (10 mg/kg) group, (3) SiO_2_ + PBS group, and (4) SiO_2_ + Trig (10 mg/kg) group. SiO_2_ (200 mg/kg in 50 mg/mL PBS) or PBS was administered for endotracheal perfusion, as previously described [32]. The starting day of the model was designated as day 1. Starting on day 28, mice in the PBS + Trig (10 mg/kg) group and SiO_2_ + Trig group were administered Trig (10mg/kg) intraperitoneally for 14 consecutive days. Meanwhile, mice in the SiO_2_ + PBS group and the PBS + PBS group received an equal amount of PBS. On day 42, the mice were euthanized to analyze the pulmonary fibrosis phenotype (Fig. 1B).

The BLM-induced fibrosis model involved randomly dividing all mice into four groups: (1) PBS + PBS group, (2) PBS + Trig (10 mg/kg) group, (3) BLM + PBS group, and (4) BLM + Trig (10 mg/kg) group. Pulmonary fibrosis was induced using a high-pressure nebulizing needle (BJ-PWM; BioJane Trading Ltd., Shanghai, China) with 1.5 mg/kg BLM in 50μL PBS by intratracheal injection, as previously reported [33]. Mice receiving equal amounts of PBS served as controls. The starting day of the model was set as day 1. Starting from day 14, mice in the PBS + Trig (10 mg/kg) and BLM + Trig (10 mg/kg) groups were intraperitoneally administered Trig (10 mg/kg) once a day for 7 days. Meanwhile, mice in the BLM + PBS group and the PBS + PBS group received equal amounts of PBS. On day 21 after BLM exposure, mice were euthanized to assess pulmonary fibrosis (Fig. 1C).

### Human lung tissue

Specimens were collected from patients who underwent surgical resection of lung tissues at Tongji Hospital, Tongji Medical College, Huazhong University of Science and Technology. The patients had a clinical diagnosis of a lung mass that required investigation, no fibrosis was observed on imaging, and postoperative pathological diagnosis confirmed a benign lesion. Normal lung tissues greater than 5 cm distal to the site of the lesion were retained. Informed consent was obtained from the patients after the study received approval from the Ethics Committee of Tongji Hospital (TJ-IRB20231297).

Human lung tissue specimens were aseptically transferred to EP tubes, dissected from the trachea into pieces. The pieces were then spread onto 10 cm petri dishes and cultured using high-sugar DMEM medium supplemented with 10% Gibco serum. The dominant cells in the culture were human lung tissue fibroblasts. To identify the primary human lung fibroblasts, fibroblast specific protein 1 (FSP1) immunostaining was conducted, and positive results indicated that they were fibroblasts (Supplementary Fig. 1).

### Western blot analysis

RIPA lysis buffer (Beyotime, Shanghai, China) was used to homogenize cells and lung tissues. Protein blot analysis was performed following standard protocols [34]. Primary antibodies used were collagen 1 (Proteintech, Wuhan, 1:1,000), fibronectin (ABclonal, Wuhan, 1:1,000), α-smooth muscle actin (Cell Signaling Technology, USA, 1:1,000), and GAPDH (Proteintech, Wuhan, 1:1,000), Smad2/3 (Cell Signaling Technology, USA, 1:1,000), p-Smad2 (ABclonal, Wuhan, 1:1,000), p-Smad3 (ABclonal, Wuhan, 1:1,000), Akt(Cell Signaling Technology, USA, 1:1,000),p-Akt(Cell Signaling Technology, USA, 1:1,000), mTOR(ABclonal, Wuhan, 1:1,000),p- mTOR (ABclonal, Wuhan, 1:1,000),p85(Cell Signaling Technology, USA, 1:1,000) andp-p85(Cell Signaling Technology, USA, 1:1,000). A chemiluminescent substrate system was used for detection, and ImageJ software was used to analyze the gray values.

### Quantitative RT-PCR assays

RNA was extracted from cells and lung tissues using TRIzol reagent (Takara, Dalian, China). The yield and quality of the RNA samples were detected using a NanoDrop2000 spectrophotometer (ThermoScientific). Subsequently, cDNA synthesis was performed using HiScript-III-RT SuperMix (Vazyme, \Wuhan). Quantitative RT-PCR analysis was performed on a CFX96 RT-PCR Detection System (Bio-Rad) using SYBR qPCR Master Mix (Vazyme). The cycling conditions applied were an initial denaturation for 30 s at 95 °C, followed by 40 cycles of 5 s at 95 °C and 30 s at 60 °C each. Normalization of data was performed using the Ct method, presenting the average of normalized transcript levels. Actb RNA levels were used as a reference for data normalization. The following primers were used for PCR amplification: mouse-*Fn1* (5’-GTG TTT TCC ACG GAT GCT G-3’, 5’-TGC CCA CTG CTG ACT TAG G-3’), mouse-*col1a1* (5’-GCG AGT GCT GTG CTT TCT G-3’, 5’-AGG ACA TCT GGG AAG CAA A-3’), and mouse-*Actb* (5’-TAT GCT CTC CCT CAC GCC A-3’, 5’-CGC ACG ATT TCC CTC TCA G-3’).

### Cellular immunofluorescence

The fibroblasts were fixed with 4% formaldehyde for 5 min and washed three times with PBS. Next, the cells were exposed to a 10% normal donkey serum solution in PBS for 1 h to block non-specific binding. Subsequently, the cells were incubated overnight at 4 °C with primary antibodies against Collagen 1 (1:100), α-SMA (1:100), and Fibronectin (1:100). After primary antibody incubation, the cells were subjected to the corresponding secondary antibody dressing. Finally, the cell nuclei were stained with DAPI and observed under an Olympus fluorescence microscope.

### Immunohistochemical analysis

The left lung was perfused with 4% paraformaldehyde via endotracheal infusion for 24 h. It was then paraffin-embedded and cut into 5μm sections. The sections were stained using established techniques, including H&E staining, Sirius red, and Masson staining [35]. For immunofluorescence staining, the sectioned tissues were co-incubated using antibodies to Collagen 1 (1:100), α-SMA (1:100), and Fibronectin (1:100), followed by incubation of the fluorescent secondary antibody, and photographs were taken using an Olympus fluorescence microscope.

### Cell migration

Fibroblasts were cultured in tissue culture dishes with appropriate medium and 10% fetal bovine serum until they reached 70–80% density. The cells were then pretreated with medium containing 1% serum. A 200μL pipette tip was used to create a cross-shaped linear scratch in the cell culture dish. The changes were recorded under a microscope at 0, 12, and 24 h and analyzed using ImageJ software.

### EdU assay

The impact of Trig on fibroblast proliferation was investigated utilizing the Cell Bright EdU-Apollo488 in vitro kit (RiboBio), following previously established protocols [36]. In brief, fibroblasts underwent treatment with pbs, L-Trig (40 μM), or H-Trig (80 μM) for a duration of 24 h. Then, cells were exposed to 100 μL of EdU solution (50 μM) for 2 h, followed by fixation with formaldehyde (4%) for 30 min. Subsequently, fluorescently labeled Apollo488 solution was added to the reaction mixture and incubated for another 30 min. Finally, nuclei were counterstained with DAPI and visualized under a microscope (Leica Microscopy, Germany).

### Statistical analysis

Statistical differences were compared using Prism software (Prism 9; GraphPad Software, CA, USA). When comparing multiple groups, we used one- or two-way analysis of variance (ANOVA) combined with Tukey's multiple comparisons test for data that conformed to normal distribution. All experiments were performed with at least three independent replications. The data are expressed as mean ± standard deviation. *p* < 0.05 was considered statistically significant.

## Result

### Trig treatment alleviated pulmonary fibrosis in silicosis

**Fig. 2:**
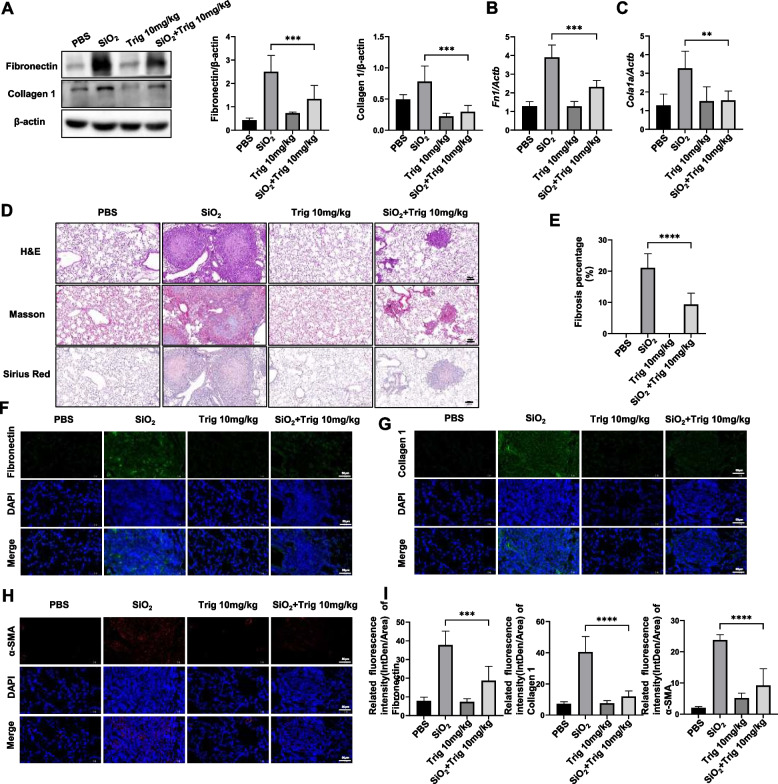
Trig attenuates silica-induced pulmonary fibrosis in mice. **A** Western blot analysis of fibronectin, collagen 1 after Trig intervention. Left panel: representative western blot results. Right panel: bar graph of western blot results; (**B**, **C**) RT-PCR analysis of *Fn1* (**B**) and *Col1a1* (**C**) expression. **D** Representative images for H&E (top), Masson's stain (middle) and Sirius red (bottom) in mice after BLM induction. **E** A bar graph showing the fibrosis foci; (**F-I**) representative immunofluorescence images and analysis of fibronectin (**F**), collagen I (**G**) and α-SMA (**H**) in lung tissue sections, magnification × 400. 5–6 mice per group. **p < 0.01, *** *p* < 0.001, **** *p* < 0.0001

To assess whether Trig could effectively attenuate SiO_2_-induced pulmonary fibrosis, we constructed a mouse model of silicosis. The silicosis mouse model was induced by intratracheal instillation of SiO_2_ suspension for 6 weeks. And we initiated the oral administration of Trig (10mg/kg) or PBS to each mouse from day 28 and continued until day 42 (Fig. 1B). Subsequently, lung tissues were collected and assessed for the pathophysiological parameter of pulmonary fibrosis. The results have shown that Trig-treated group manifested a significant reduction in the levels of these fibrotic markers (Fig. 2A). Furthermore, mRNA expression levels were consistently downregulated by Trig therapy (Fig. 2 B and C). The H&E, Masson and Sirius Red histological staining of mouse lung tissues revealed significant structural damage to the the SiO_2_ group’s lung tissue structure, which is exhibited by increased thickness of the alveolar septa and thickening of both alveolar and bronchial walls, as well as increased thickness of alveolar septa. Furthermore, significant inflammatory cell infiltration in the alveolar gaps and increased collagen fiber deposition were noted, both of which are suggestive of worsened pulmonary fibrosis. Remarkably, Trig application resulted in marked improved lung tissue inflammation and fibrosis compared to the SiO_2_ group. (Fig. 2D and G). We examined the expression of fibrosis-related markers, such as α-SMA, Collagen1, and fibronectin, as measured by immunofluorescence in lung tissues, in order to confirm the degree of fibrosis improvement. The results indicated that the expression of fibrosis markers was lower in the Trig + SiO_2_ group than SiO_2_ group (Fig. 2F-I).

### Trig administration attenuated BLM-induced pulmonary fibrosis

**Fig. 3:**
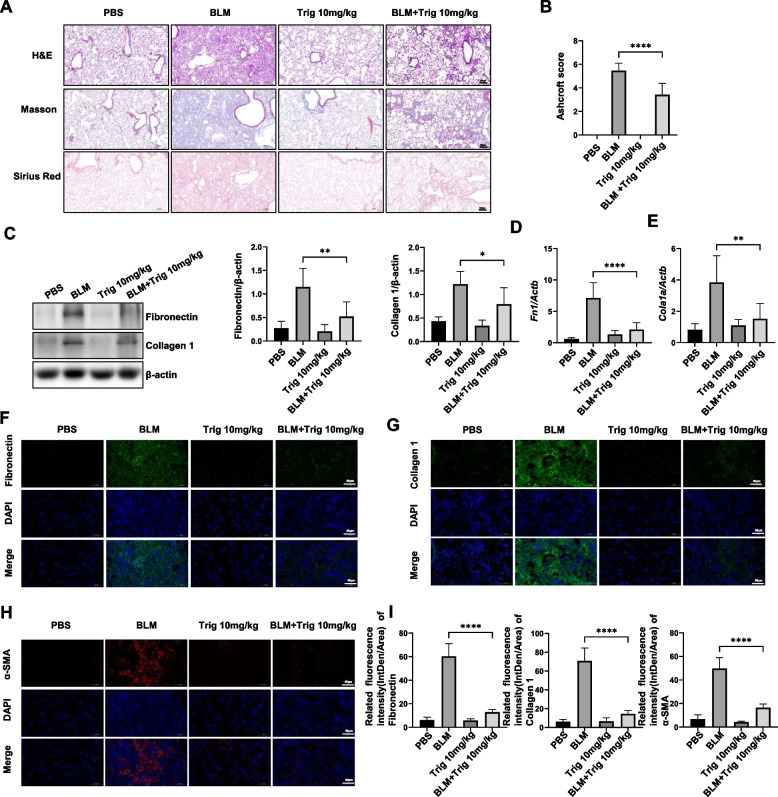
Trig ameliorated BLM-induced pulmonary fibrosis in mice. **A** Representative images for H&E (top), Masson's stain (middle) and Sirius red (bottom) in mice after BLM induction. Magnification × 200; (**B**) Ashcroft scores for the severity of fibrosis; (**C**) Western blot of fibronectin, collagen 1 expression after Trig infection; Left panel: representative western blot results. Right panel: bar graph of western blot results; (**D**, **E**) RT-PCR analysis of *Fn1* (**D**) and *Col1a1* (**E**) expression. expression expansion; **F-I** Representative immunofluorescence images and analysis of fibronectin (**F**), collagen I (**G**) and α-SMA (**H**) in lung tissue sections. Magnification × 400. 5–6 mice per group. *p < 0.05, **p < 0.01, **** *p* < 0.0001

To further validate the efficacy of Trig in the treatment of pulmonary fibrosis in vivo, another typical and mature pulmonary fibrosis model was applied. Mice were given intratracheal injections of BLM to cause pulmonary fibrosis. After 7 days of treatment with 10 mg/kg Trig starting on day 14, samples were taken on day 21 (Fig. 1C). H&E staining of pathological tissues presented that the extent of lung injury was remarkably reduced in the Trig intervention group compared to the control group. Sirius red and Masson's staining of collagen fibers revealed a markedly attenuated degree of collagen deposition in Trig group mice compared to control mice (Fig. 3A and B). Furthermore, the protein and mRNA expression levels of fibrosis related makers in lung tissues stimulated by BLM were vastly attenuated in the Trig + SiO_2_ group, (Fig. 3C-F). Similarly, immunofluorescence staining analysis research that Trig therapy might lessen the degree of fibrosis, as seen by decreased α-SMA, Collagen1, and fibronectin fluorescence intensity (Fig. 3G-K). Collectively, our study provided compelling evidence of the therapeutic effect of Trig in BLM-induced lung injury and fibrosis.

### Trig suppressed fibroblast to myofibroblast differentiation

The differentiation of fibroblasts into myofibroblasts is a crucial factor in the progression of pulmonary fibrosis [37]. To investigate the effect of Trig on fibroblast activation, we first pre-stimulated human primary fibroblasts with Trig for 2h, followed by stimulation with TGF-β1 for 24 h while administering different concentrations of Trig. Notably, doses of 40 µM were categorized as the L-Trig group, while 80 µM doses were designated as the H-Trig group. As expected, TGF-β1 stimulation resulted to an increase in the protein expression of α-SMA, a myofibroblast activation marker.

**Fig. 4:**
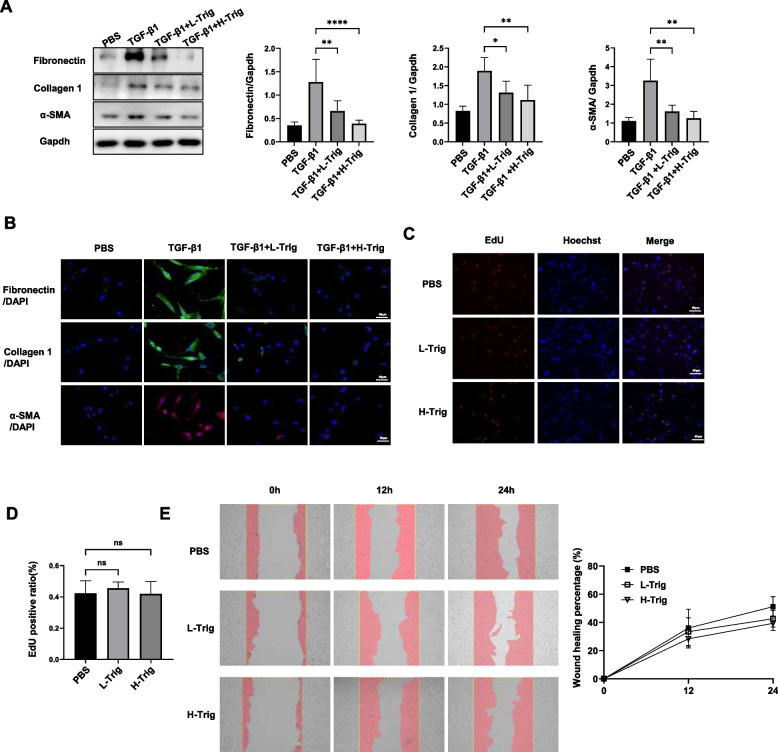
Effect of Trig on fibroblast activation, proliferation and migration. **A** WB analysis of fibronectin, collagen 1 and α-SMA expression in fibroblasts. Left panel: representative western blot results. Right panel: bar graph of the western blot results; (**B**) Immunofluorescence of fibronectin, collagen 1 and α-SMA in fibroblasts; (**C**) Representative data in fibroblasts with EdU staining; (**D**) Histogram data of EdU staining; (**E**) Representative data of fibroblast scratch assay. L-Trig: 40 µM /mL of Trig, H-Trig: 80 µM /mL of Trig. * *p* < 0.05, **p < 0.01, **** *p* < 0.0001

Additionally, fibrotic proteins such as fibronectin and collagen 1 were highly up-regulated, indicating that the fibroblasts were in an activated state and accompanied by ECM deposition (Fig. 4A). Interestingly, after Trig intervention, there was a significant concentration-dependent inhibition of fibroblasts' ability to differentiate into myofibroblasts (Fig. 4A). The cell immunofluorescence staining provided additional proof of this phenomenon (Fig. 4B). We further examined the effect of Trig on fibroblast migration and proliferation. However, our analysis of EdU staining revealed minimal variation in the number of double-positive cells between the Trig and PBS groups (Fig. 4C and D). Similarly, cell migration assessment indicated that Trig administration didn’t decrease wound healing ability (Fig. 4E). Consequently, these data demonstrated that Trig significantly reduces pulmonary fibrosis by attenuating the conversion of fibroblasts to myofibroblasts, but without affecting cell migration and proliferation.

### Trig mitigated pulmonary fibrosis through inhibition of TGF-β/Smad signaling

**Fig. 5:**
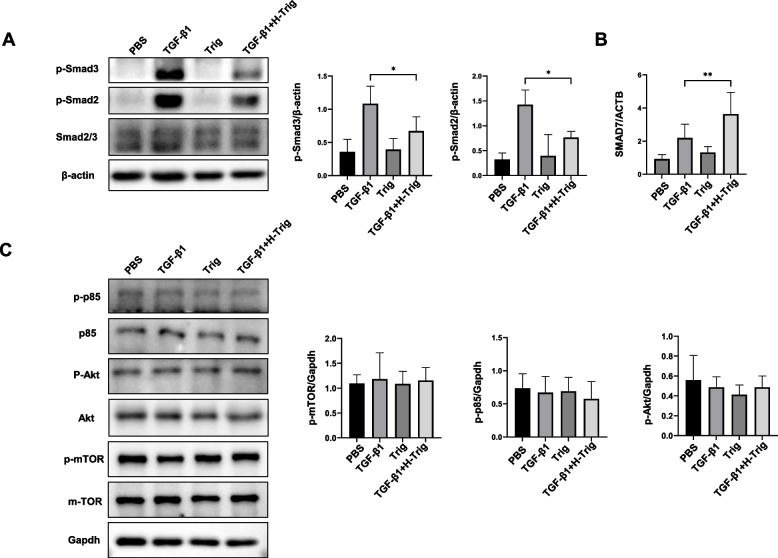
Impacts of Trig on TGF-β-stimulated Smad signaling. **A** Trig reduced TGF-β-induced Smad signaling. Left panel: representative western blot result for p-Smad2, p-Smad3, Smad2/3 at 1 h TGF-β stimulation. Right panel: figures showing the data with three replicates **B** RT-PCR analysis of SMAD 7expression. **C** Trig had no effect on PI3K/AKT signaling pathway. * *p* < 0.05

The process of fibroblast to myofibroblast differentiation encompasses a complex interplay of various signaling pathways [38]. By activating Smad-dependent or independent pathways, TGF-β plays a crucial role in fibroblast activation and consequently contributes to the development of fibrotic diseases. Upon interaction with its receptor complex, TGFβR, TGF-β facilitates the phosphorylation and activation of Smad2 and Smad3 proteins. These proteins then ally with the co-Smad4, forming a complex that migrates to the nucleus to influence transcriptional regulation [39]. Therefore, we assessed the effect of Trig on TGF-β/Smad pathway signaling in TGF-β1-stimulated primary lung fibroblasts. After TGF-β1 stimulation, The TGF-β/Smad pathway was markedly activated, as evidenced by elevated levels of p-Smad2 and p-Smad3 in primary lung fibroblasts. Treatment with Trig significantly increased Smad 7 and reduced the levels of p-Smad2 and p-Smad3 (Fig. 5A). Beyond the activation of this classical Smad pathway, TGF-β also engages alternative signaling mechanisms, notably through the activation of the PI3K/AKT pathway [39]. Nevertheless, there was no discernible change in the PI3K/AKT pathway, which includes p-mTOR, p-p85, and p-Akt (Fig. 5B). Overall, Trig modulates the differentiation of fibroblasts into myofibroblasts by attenuating the phosphorylation of smad2/3 in the TGF-β1/Smad pathway, while exhibiting no inhibitory effect on the PI3K/AKT pathway.

### Toxicity assessment of Trig in the treatment of pulmonary fibrosis

**Fig. 6:**
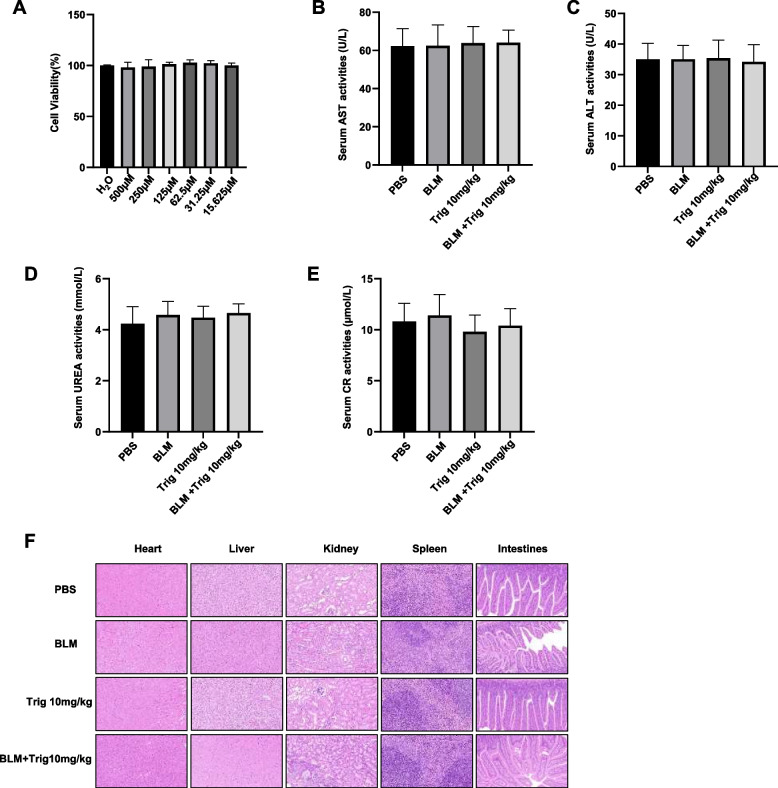
Toxicity and safety analysis of Trig as a therapeutic drug. **A** cytotoxicity analysis; (**B**, **C)** AST, ALT representing liver function; (**D**, **E)** CR, UREA representing renal function; (**F**) H&E staining of various organ tissues: The sequence begins with heart tissue (far left), progresses to liver tissue (second from the left), centers on kidney tissue, and concludes with sections of intestine (first from the right) and spleen tissue (far right)

Based on the aforementioned results, Trig has considerable potential for the treatment of pulmonary fibrosis in silicosis. Subsequently, we conducted comprehensive assessments to evaluate the safety profile of Trig both in vitro and in vivo. In vitro studies using the CCK8 essay found that Trig at up to 500 μM, which is more than six times the intervention concentration, had no meaningful effect on cell viability (Fig. 6A). In addition, in vivo testing included the collection of heart, liver, intestine, spleen, kidney and serum samples from Trig-treated mice after 21 days. Surprisingly, biochemical analysis of serum samples confirmed that efficacious concentrations of Trig didn’t impair liver (AST and ALT) or kidney (CR and UREA) function (Fig. 6B-E). Moreover, histological examination by H&E staining revealed no discernible pathological changes in the heart, liver, spleen, kidney or intestine. (Fig. 6F). In summary, these results highlight the favorable safety profile of Trig and highlight its potential as a promising therapeutic candidate for further exploration.

## Discussion

**Fig. 7:**
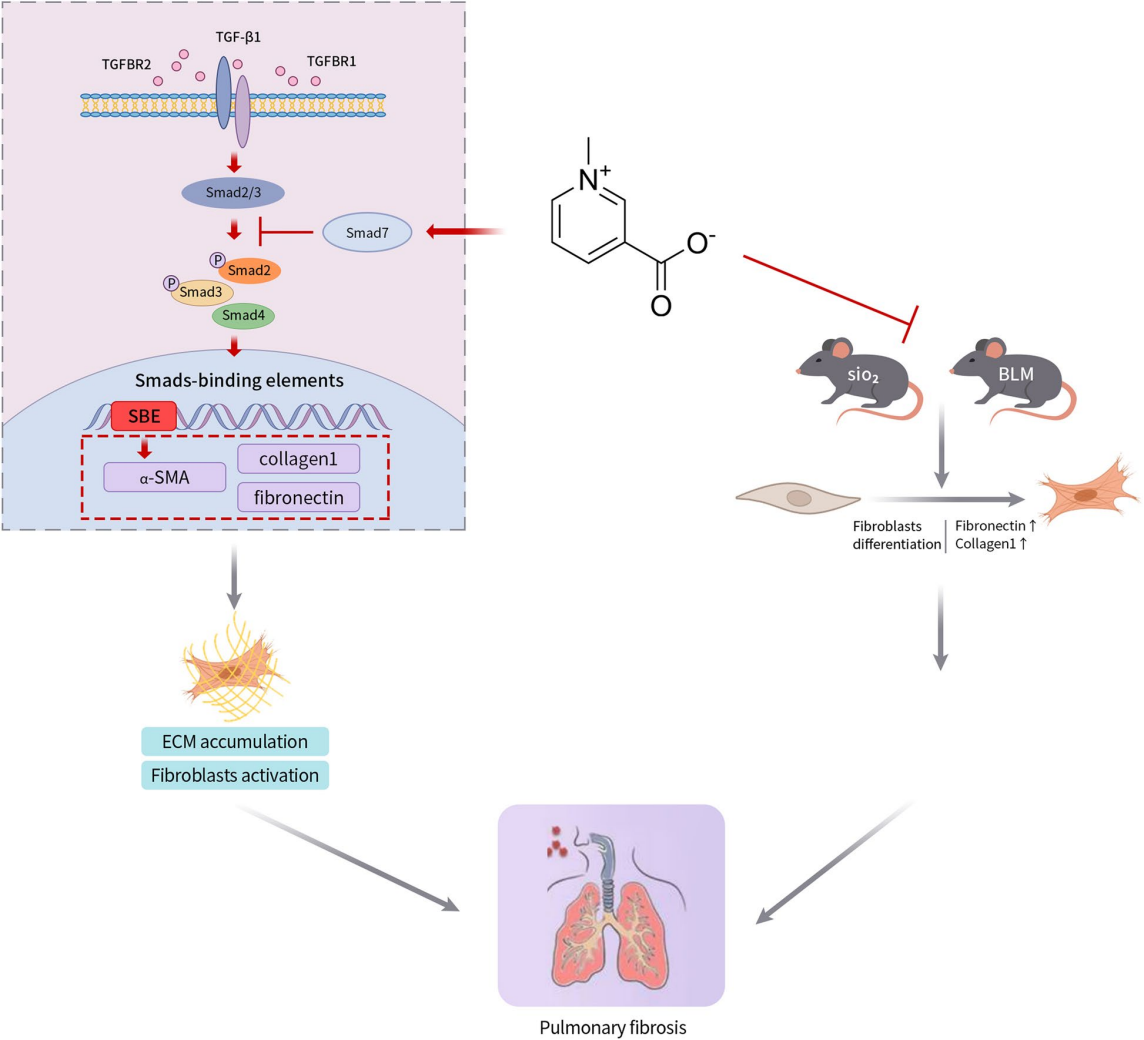
Schematic diagram of the mechanism by which Trig affects pulmonary fibrosis. Trig attenuates pulmonary fibrosis by inhibiting the TGF-β/Smad signaling pathway in vitro, reducing the differentiation of fibroblasts to myofibroblasts and decreasing ECM deposition

Silicosis, an interstitial lung disease caused by the inhalation of crystalline silica particles, is characterized by acute and chronic inflammation, followed by diffuse fibrosis, ultimately leading to progressive respiratory insufficiency. Currently, it is one of the most dangerous occupational diseases worldwide, with an estimated 10,000 or more new deaths per year [40]. However, the treatment for silicosis has been in a state of scarcity, so it’s urgent to explore new and effective treatment means. In the present investigation, we demonstrated that therapeutic effects of Trig in mitigating pulmonary fibrosis caused by silica and impeding the transformation of fibroblasts to myofibroblasts. The potential mechanism for protective of Trig in silicosis-related pulmonary fibrosis may be attributed to the inhibition of the TGF-β/Smad signaling pathway (Fig. 7). These findings advocated that Trig might be a valuable therapeutic approach for silicosis fibrosis.

Trig is a phytobasic alkaloid extracted from coffee and fenugreek, which possesses a variety of biological activities [28]. It has been reported to execute therapeutic effects against inflammatory, oxidant [25], and anticancer effects [41]. Moreover, it can alleviate renal fibrosis by upregulating the TGF-β1 signaling inhibitor [29] and suppress high glucose-induced mesangial cell proliferation and ECM expression by modulating the Wnt signaling pathway [42]. After prolonged exposure to silica dioxide inhalation, silicosis manifests as a fibrotic disorder characterized by mechanisms including oxidative stress, cytokine and chemokine production, and collagen accumulation [23].These observations prompt our consideration of Trig's potential as a therapeutic option for treating silicosis fibrosis.

To evaluate the therapeutic efficacy of Trig in managing silicosis fibrosis, we adopted a silica-induced pulmonary fibrosis animal model. Surprisingly, Western blot and RT-PCR validation revealed reduction in fibrosis markers of Trig + SiO_2_ group, including Fibronectin and Collagen 1. Furthermore, the effectiveness of Trig in reducing silica-induced pulmonary fibrosis was confirmed by immunofluorescence analysis combined with histological staining using H&E, Masson's trichrome, and Sirius Red. According reported, pulmonary fibrosis can be caused by a plethora of etiological factors, involves a common pathogenic pathway characterized by the activation of fibroblasts, which in turn precipitates the accumulation of the ECM [38, 43]. Based on this, we also investigated the feasibility of Trig in BLM-associated pulmonary fibrosis. Naturally, Trig is a very attractive option for the treatment of fibrosis in the setting of BLM.

Although Trig is forceful in preventing and attenuating silica-induced pulmonary fibrosis, the underlying mechanisms are unknown. In silicosis, pulmonary fibrosis arises from aberrant cellular repair of alveolar epithelial injury, wherein repetitive stimuli induce persistent damage to alveolar epithelial cells and subsequent release of substantial cytokines, facilitating the differentiation of fibroblasts into myofibroblasts and instigating an abnormal accumulation of ECM [44, 45]. In our investigation, we illustrate that the treatment of Trig effectively attenuated the differentiation of fibroblasts into myofibroblasts induced by TGF-β1.

The dysregulation of the TGF-β1/Smad pathway plays an essential role in the pathological process of silicosis [44, 46]. TGF-β1 recruits and activates transforming growth factor beta receptor 1(TGFR1) upon binding to TGF-β receptor 2 (TGFβR2). Subsequently, TGFR1 is activated and phosphorylates Smad2 and Smad3. They combine with Smad4 to form the Smad complex, which translocates to the nucleus where it activates target gene expression and causes the extracellular matrix proteins to be produced continuously. Major downstream mediators Smad2 and Smad3 are essential for fibroblast activation and the pathophysiology of fibrosis [47]. This raises the question of whether Trig has an influence on TGF-β/Smad signaling. Exogenous TGF-β1 significantly increased the levels of Smad2 and Smad3 phosphorylation, but the administration of Trig upregulated Smad7, a negative regulator of the TGF-β1 signaling pathway, and inhibited TGF-β1-induced Smad2/3 phosphorylation. Although the PI3K/AKT signaling pathway is also involved in silicosis pulmonary fibrosis [48]. No conspicuously differences were detected in the phosphorylated forms of PI3K, Akt, and mTOR. This implies that the consequences of fibroblast differentiation could not be mediated via the PI3K/AKT signaling pathway. The efficacy of Trig in attenuating bleomycin-induced pulmonary inflammation and fibrosis through modulation of the NLRP3 inflammasome and Hippo signaling pathway had been reported [49]. We also validated this cardiac signaling pathway in mouse models induced by silica and BLM. Notably, distinct alterations in the signaling pathway were observed exclusively in the bleomycin-induced pulmonary fibrosis model, while no significant differences were detected in the silica-induced pulmonary fibrosis model (see Supplementary Fig. 2).

It is critical that Trig is safe both in vitro and in vivo. We examined biochemical parameters in serum, including ALT, AST, CR and UREA. Furthermore, H&E staining changes in heart, spleen, kidney and liver also assessed. No organ damage was observed from the continuous intraperitoneal delivery of Trig, as indicated by the lack of fluctuation in the previously described parameters in any of the experimental groups. Taken together, Trig may be could be regarded as safe for use in therapeutic settings.

In conclusion, this study indicates that Trig can reverse silicosis pulmonary fibrosis. Additionally, Trig inhibits the differentiation of fibroblasts to myofibroblasts by inhibiting the TGF-β/Smad signaling axis, making it a safe option. These findings could provide new insights into the treatment of silicosis and other pulmonary fibrosis.

### Supplementary Information

Supplementary Material 1.

Supplementary Material 2.

## Data Availability

No datasets were generated or analysed during the current study.

## Abbreviations

ALT: Alanine Aminotransferase
AKT: Protein Kinase B
AST: Aspartate Aminotransferase
CR: Creatinine Clearance Rate
ECM: Extracellular matrix
PI3K: Phosphoinositide 3-kinase
TGF-β1: Transforming growth factor-β1
TGFR1: Transforming growth factor-β receptor 1
TGFβR2: Transforming growth factor-β receptor 2
NLRP3: NOD-, LRR- and pyrin domain-containing protein 3
