# Supplementary figures and images for: Trigonelline hydrochloride attenuates silica-induced pulmonary fibrosis by orchestrating fibroblast to myofibroblast differentiation
Source: Respir Res. 2024 Jun 15;25:242. doi: 10.1186/s12931-024-02876-1 (PMC11179236; doi:10.1186/s12931-024-02876-1)

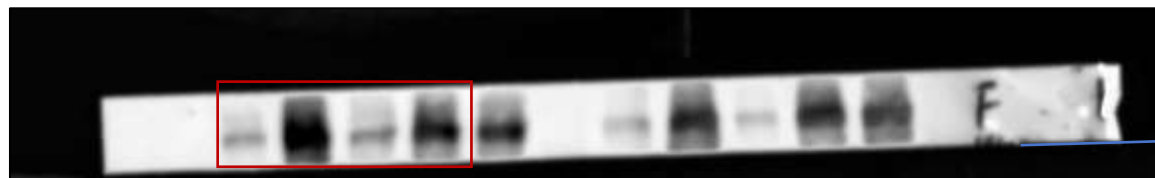

180kD

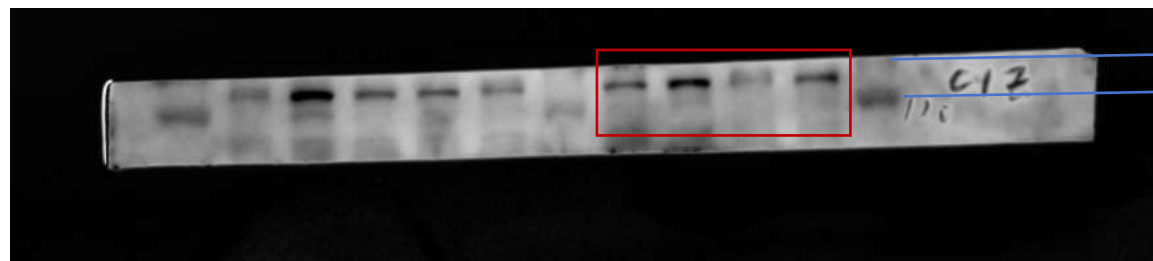

180kD

130kD

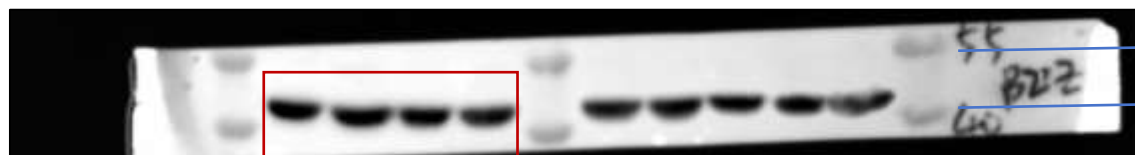

55kD

40kD

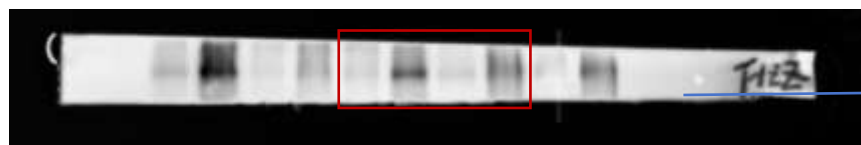

180kD

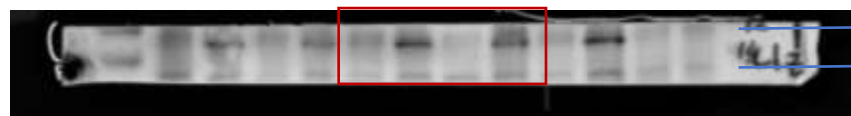

180kD

130kD

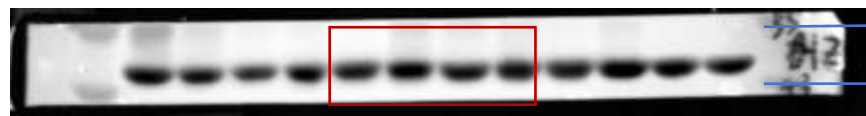

55kD

43kD

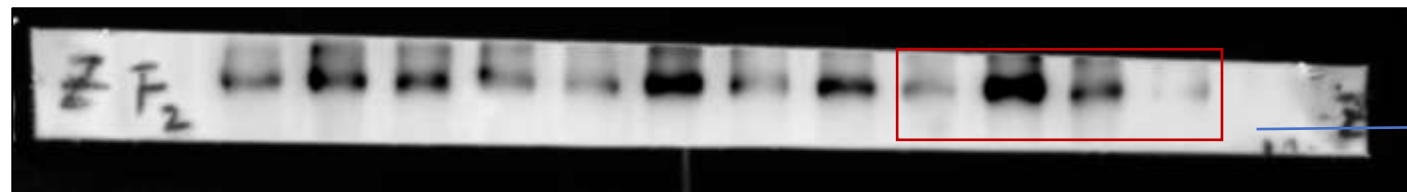

180kD

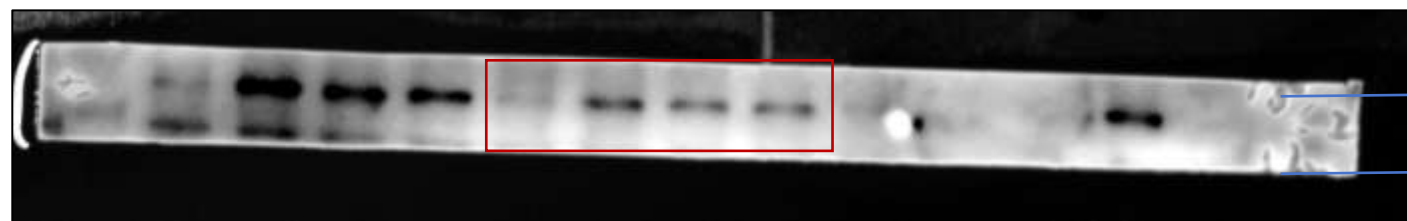

180kD

130kD

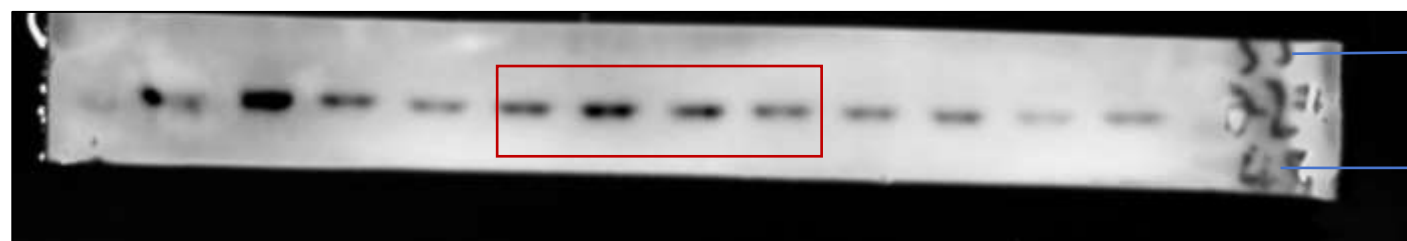

55kD

43kD

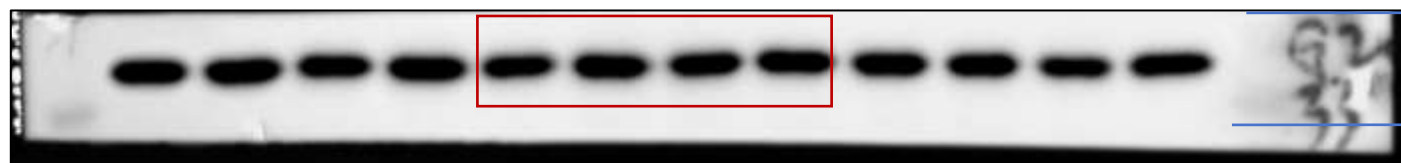

43kD

33kD

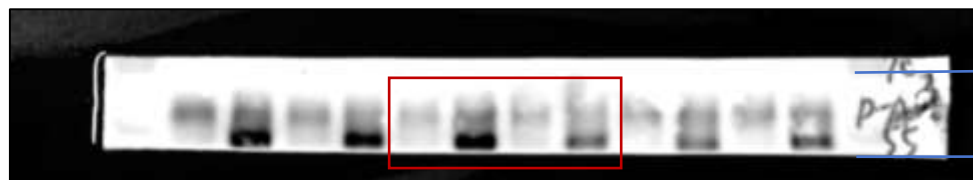

70kD

55kD

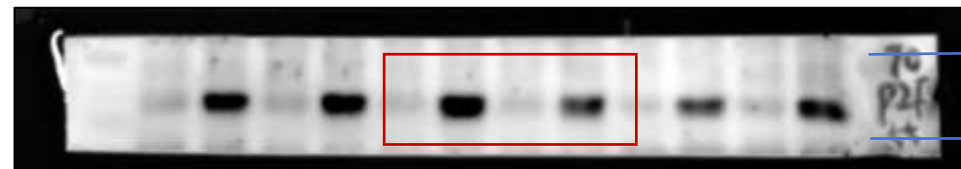

70kD

55kD

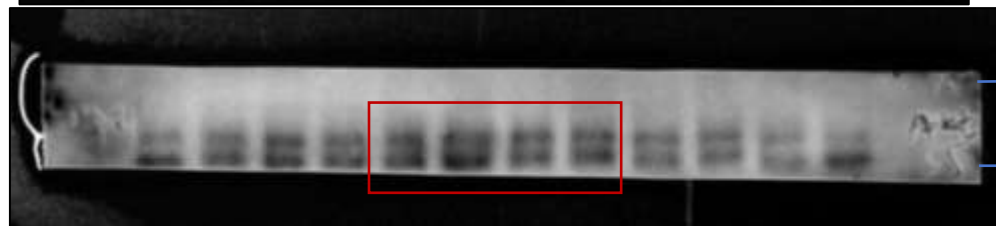

70kD

55kD

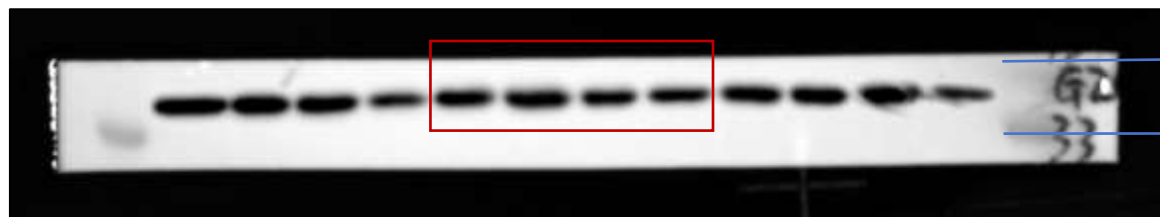

43kD

33kD

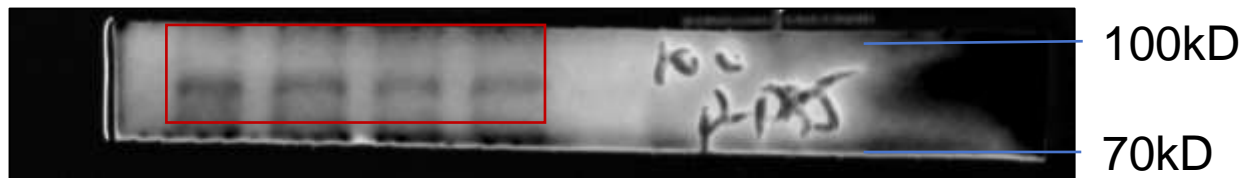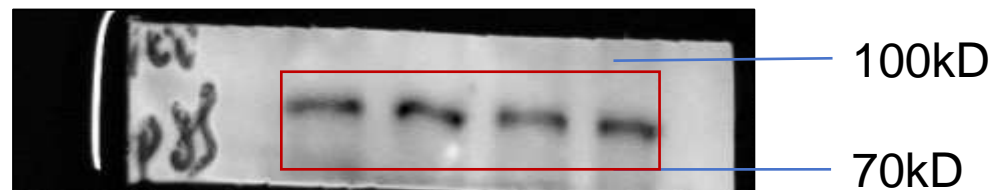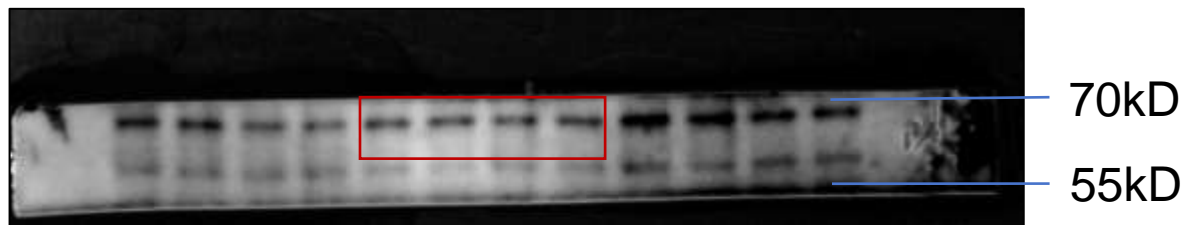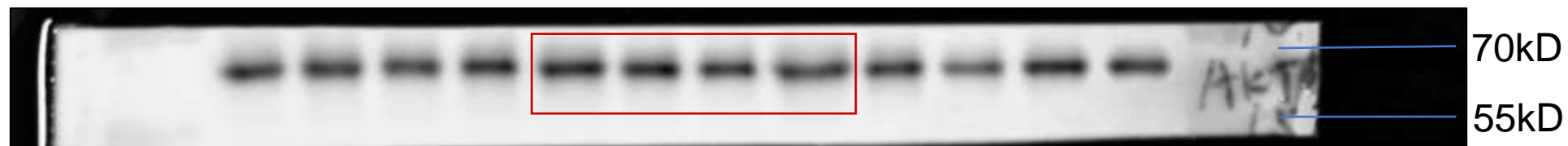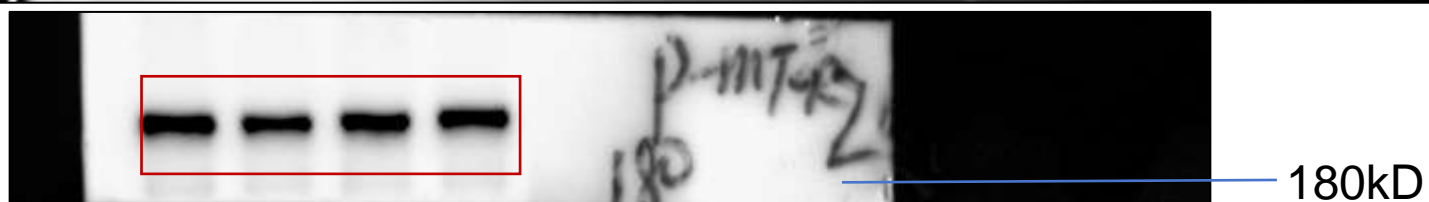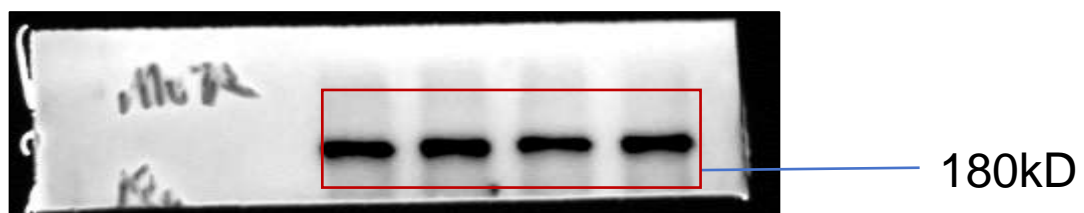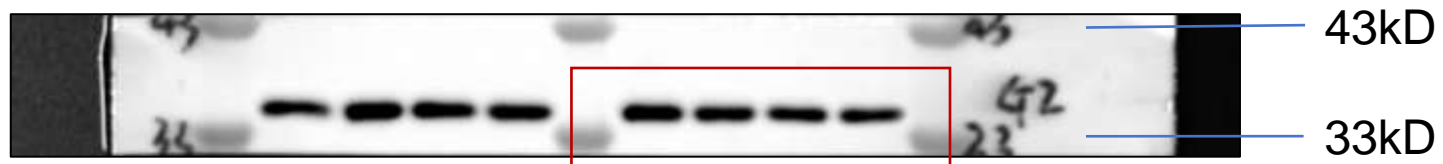

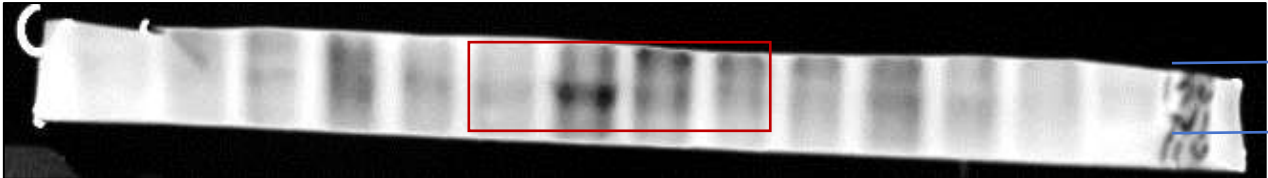

130kD

100kD

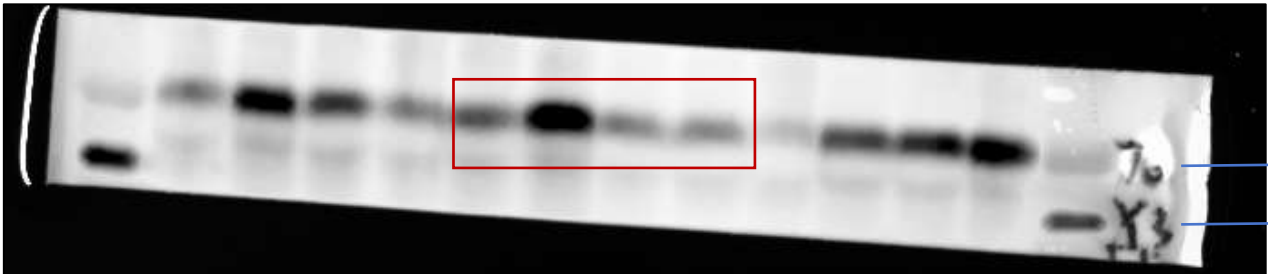

70kD

50kD

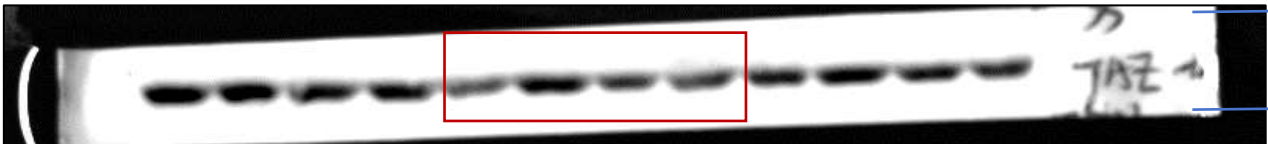

55kD

42kD

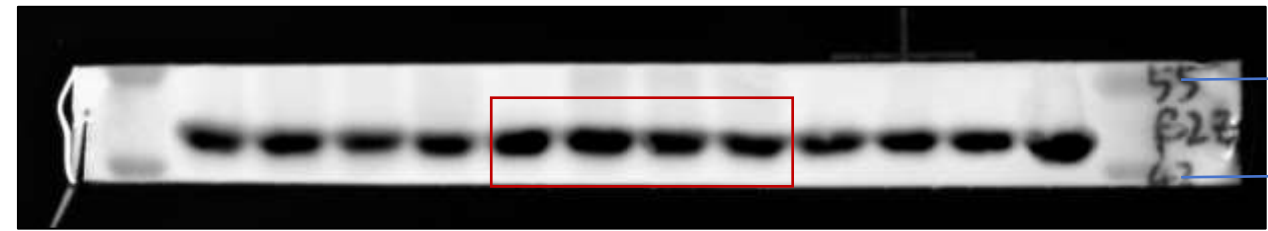

55kD

42kD

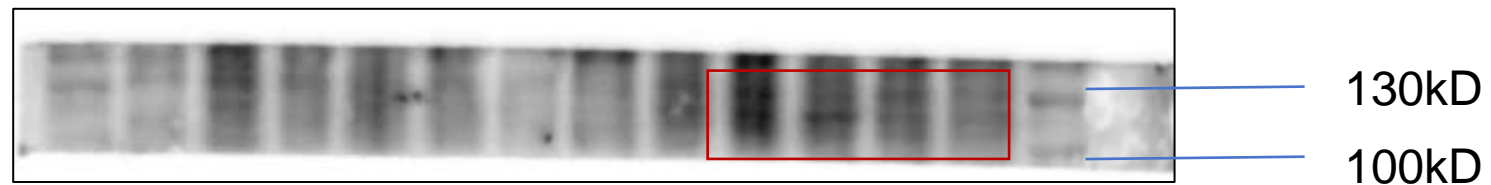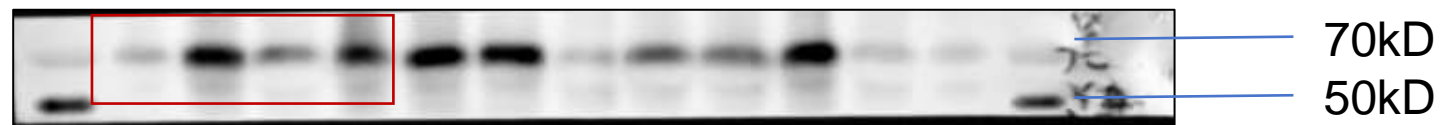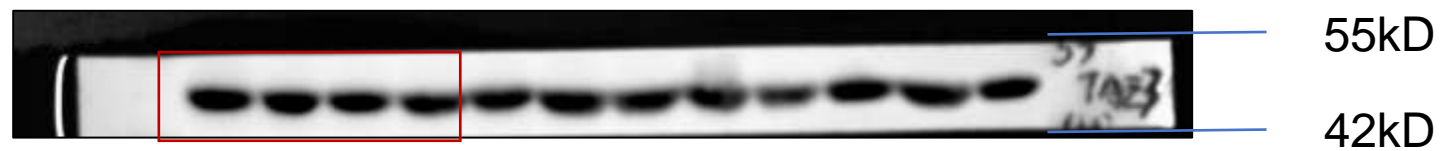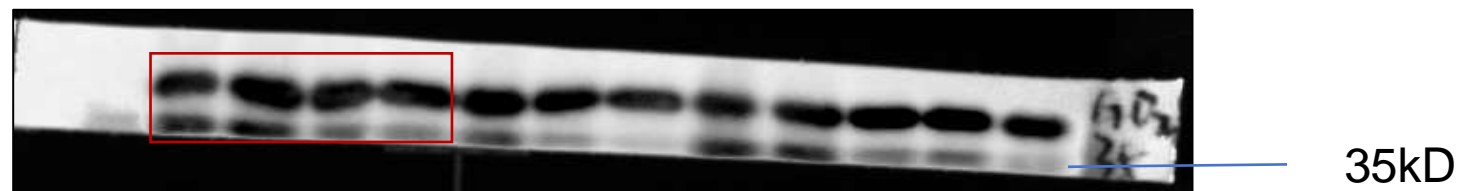

Supplement: Supplementary file 1 — Supplementary Material 1. [file 12931_2024_2876_MOESM1_ESM.pdf]
