# Supplementary material for: Trigonelline hydrochloride attenuates silica-induced pulmonary fibrosis by orchestrating fibroblast to myofibroblast differentiation
Source: Respir Res. 2024 Jun 15;25:242. doi: 10.1186/s12931-024-02876-1 (PMC11179236; doi:10.1186/s12931-024-02876-1)
Supplement: Supplementary file 2 — Supplementary Material 2. [file 12931_2024_2876_MOESM2_ESM.pdf]

A

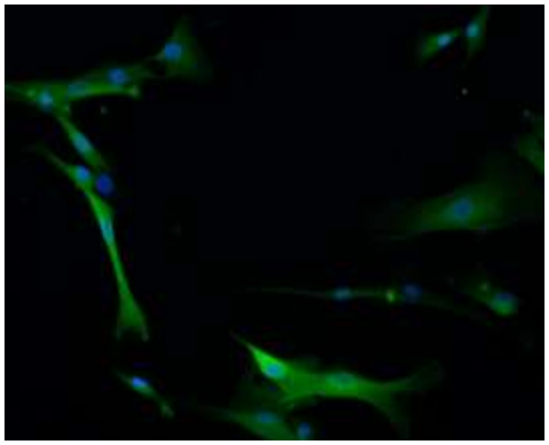

B

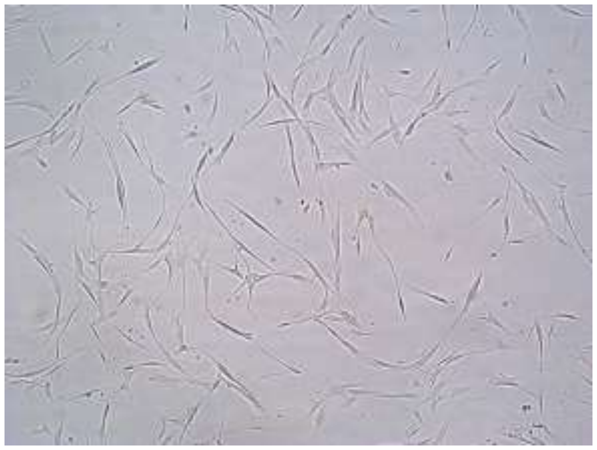

Supplementary Figure 1 Identification and Analysis of Fibroblasts: A: Fibroblasts under light microscopy, (50× magnification); B: FSP1 staining of fibroblasts, (400× magnification).

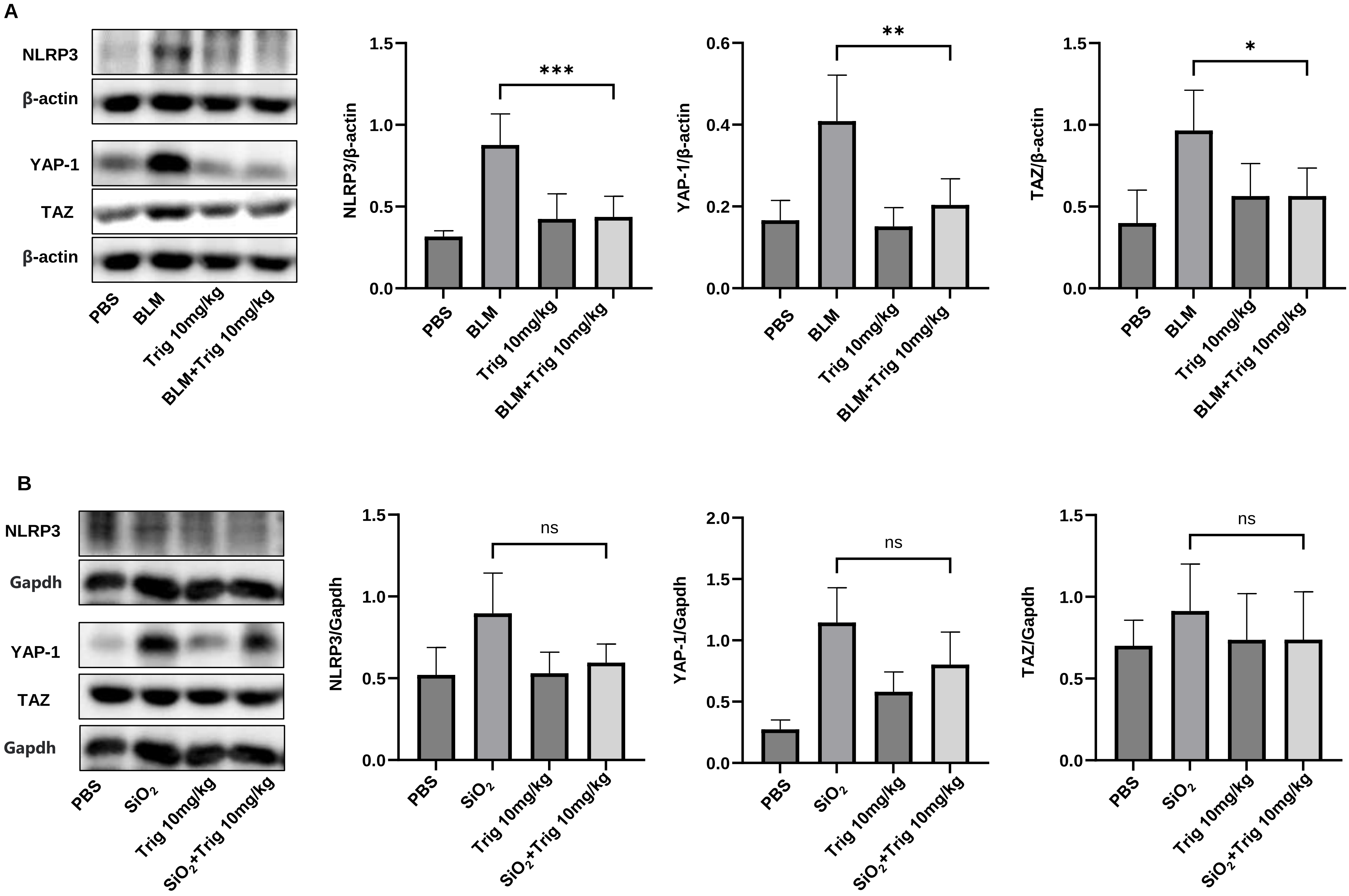

Supplementary Figure 2 The effects of Trig on NLRP3 inflammasome and Hippo signaling in BLM-induced and silica-induced mouse models. (A) The protein levels of NLRP3 inflammasome and Hippo signaling after BLM and Trig treatment. (B) The WB analysis of NLRP3 inflammasome and Hippo signaling in SiO2-induced PF model. Left panel: representative WB results for NRLP3, YAP-1, and TAZ. Right panel: the quantitative analysis of WB results. The data were presented as mean ± SD, * $p < 0.05$, ** $p < 0.01$, *** $p < 0.001$.
